# Supplementary material for: [99mTc]Tc-Labeled Plectin-Targeting Peptide as a Novel SPECT Probe for Tumor Imaging
Source: Pharmaceutics. 2022 May 6;14(5):996. doi: 10.3390/pharmaceutics14050996 (PMC9146797; doi:10.3390/pharmaceutics14050996)
Supplement: Supplementary file 1 [file pharmaceutics-14-00996-s001.zip › pharmaceutics-1676677-supplementary.pdf]

pharmaceutics

Supplementary Materials: [^{99m}Tc]Tc-Labeled Plectin-Targeting Peptide as a Novel SPECT Probe for Tumor Imaging

Jiali Gong, Lingzhou Zhao, Jiqin Yang, Meilin Zhu and Jinhua Zhao

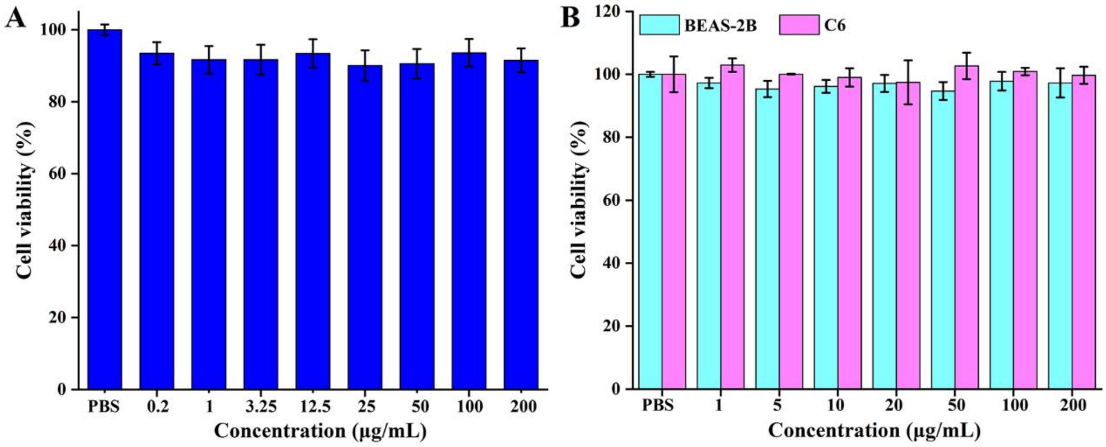

Figure S1. CCK-8 assays of (**A**) BxPC-3, (**B**) BEAS-2B and C6 cells treated with PTP at a concentration range from 0 to 200 μg/mL for 24 h.

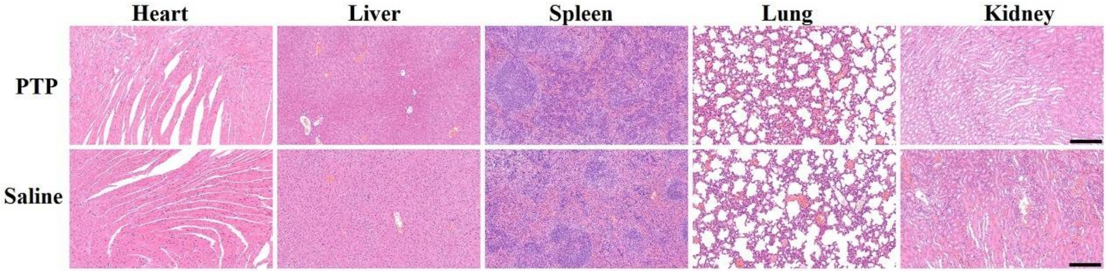

Figure S2. H&E staining results of ICR mice after treatments of PTP and saline. The scale bar represents 200 μm for all panels.

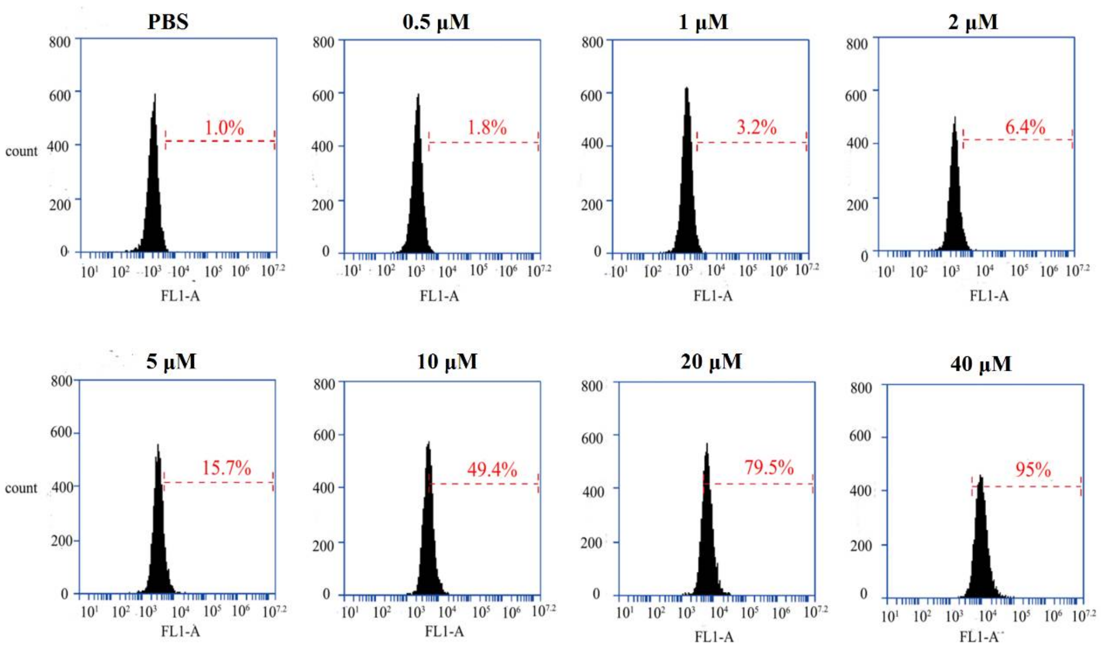

Figure S3. Flow cytometric analysis of C6 cells treated with FITC-PTP at different concentrations (0 to 40 μM) for 4 h.

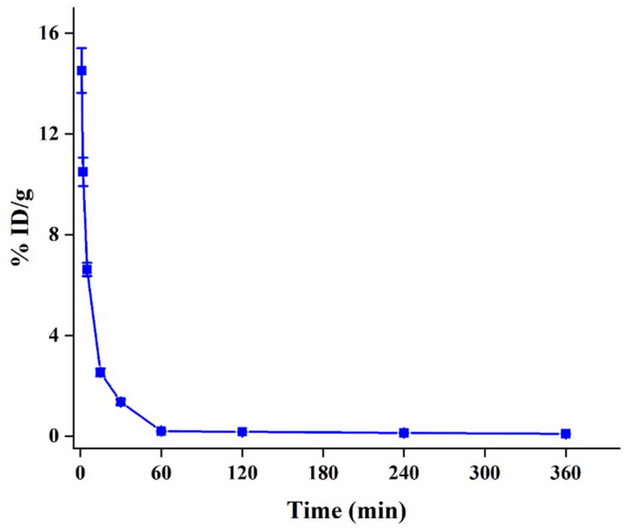

Figure S4. Radioactivity-time curve of [^{99m}Tc]Tc-HYNIC-PTP in ICR mice.

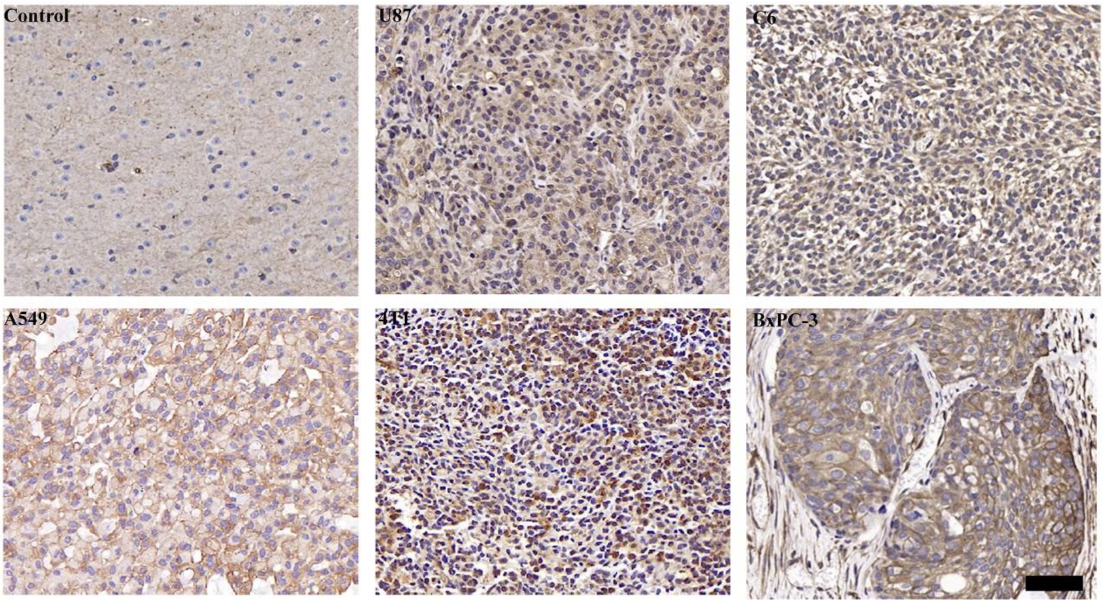

Figure S5. Immunohistochemistry images of plectin in muscle (control) and five different tumors. The relative plectin expression level was determined by calculating the proportion of positive area. The scale bar represents 100 μm for all panels.
